# Effects of different colors of plastic-film mulching on soil temperature, yield, and metabolites in *Platostoma palustre*

**DOI:** 10.1038/s41598-024-55406-w

**Published:** 2024-03-01

**Authors:** Hao Chen, Suhua Huang, Changqian Quan, Zhining Chen, Meihua Xu, Fan Wei, Danfeng Tang

**Affiliations:** 1 Guangxi Key Laboratory of Medicinal Resources Protection and Genetic Improvement/Guangxi Engineering Research Center of TCM Resource Intelligent Creation, National Center for TCM Inheritance and Innovation, Guangxi Botanical Garden of Medicinal Plants, Nanning, China; 2 National Engineering Research Center for Southwest Endangered Medicinal Materials Resources Development, Guangxi Botanical Garden of Medicinal Plants, Nanning, China; 3 College of Pharmacy, Guangxi Medical University, Nanning, China; 4 School of Traditional Chinese Pharmacy, China Pharmaceutical University, Nanjing, China

**Keywords:** *Platostoma palustre*, Plastic-film mulching, Soil temperature, Yield, Metabolites, Plant sciences, Metabolomics, Metabolomics

## Abstract

*Platostoma palustre* is an annual herb and an important medicinal and edible plant in southern China. Plastic-film mulching is an effective agronomic practice in the cultivation system of *P. palustre,* of which black-film mulching is the most common. However, fewer researches have been focused on the use of other colors of plastic films in *P. palustre* cultivation. In this study, different colors (white, black, red, and green) of plastic film were adopted, and the effects of different colors of plastic film mulching on the soil temperature, yield, and metabolites of *P. palustre* were investigated. The results showed that the fresh weight of a single plant of the green film treatment was significantly higher than that of the white film treatment (n = top 28). Based on the results of three temperature measurements, the soil temperature was almost the highest in the red film treatment and lowest in the white film treatment. The metabolomic analysis revealed that a total of 103 differential metabolites were identified. Among these, the gluconic acid, deoxyribose, and *N*-Acetylmannosamine in the red film treatment presented the highest abundance compared with the other treatments, meanwhile, the abundances of the five monosaccharides in the red film treatment were significantly higher than those of the green film treatment. Moreover, the sucrose, trehalose, and D-(+)-trehalose in the green film treatment exhibited the highest abundance, and the abundances of eight different amino acids in the red film treatment were almost the lowest while those in the black film treatment were almost the highest. Further analysis of the membership function values indicated that the black and red film treatments might be more suitable for the cultivation and quality production of *P. palustre* in comparison with the other two treatments. This study will provide a theoretical basis for improving the efficient cultivation technology of *P. palustre* and forming a theoretical system of *P. palustre* film mulching cultivation.

## Introduction

Plastic-film mulching is a common cultivation and management technology in agricultural production. Generally, this technique plays a role in regulating soil temperature, preserving soil moisture, and shortening crop growth period, and some special films have the functions of cooling, preventing weeds, avoiding aphids, and reflecting light^1–4^. Plastic-film mulching has a certain role in improving the yield and quality of crops and has been widely used in a variety of crop cultivation, such as corn^5^, peanut^6^, citrus^7^, cucumber^8^, potato ^1^, etc.

*Platostoma palustre* is an annual herb belonging to the *Platostoma* genus of Labiatae, which is an important medicinal and edible plant in southern China^9–13^. The active ingredients of *P. palustre* mainly include polysaccharide, quercetin, oleanolic acid, ursolic acid, caffeic acid, and so on^14^, which possess multiple biological activities, like anti-hypertensive effect^15^, hypolipidemic effect^16^, hepatoprotective effect^17^, antioxidation^18^, and antibiosis^19^. Nowadays, it is widely used as a raw material in the food industry, with a wide range of uses and high market and economic value.

*Platostoma palustre* is usually transplanted in the early spring season when the temperature is low in south China, therefore plastic-film mulching is an effective agronomic measure in the cultivation system of *P. palustre.* Plastic-film mulching can be used to create good microclimate conditions under the film including heat, air, water, and fertilizer conditions suitable for the growth and development of *P. palustre*^20^*.* In the planting process of *P. palustre*, plastic-film mulching has been widely used in different production areas in China, especially the black plastic mulch is generally used for the cultivation of *P. palustre*. Studies have shown that black plastic-film mulching during the whole growth period can reduce labor intensity, lower production costs, and increase yields^21^.

Currently, different colors of plastic film are available on the market and vary in their effects of increasing temperature and moisture retention, thus affecting the growth, development, and yield of crops^22,23^. As mentioned above, the research on the plastic film mulching in *P. palustre* is mostly limited to the black plastic film, while fewer researches have been focused on the use of other colors of plastic films in *P. palustre* cultivation. In this study, to screen out the most suitable types of mulching film for *P. palustre* cultivation, we intend to adopt different colors (white, black, red, and green) of plastic film to plant *P. palustre*. The effects of different color plastic-film mulching on the yield of *P. palustre* were studied, and the differences in metabolites between different treatments were also compared and analyzed using metabolomics. This study will provide a theoretical basis for improving the efficient cultivation technology of *P. palustre* and forming a theoretical system of *P. palustre* film mulching cultivation.

## Materials and methods

### Plant materials

In this study, four plastic-film mulching treatments in different colors (red, green, black, and white) were employed to cultivate *P. palustre* at the Lingshan region in Guangxi, China. On April 21, 2022, *P. palustre* cuttage seedlings were transplanted into the field with normal field management practices. The ridge had a length of 9 m, width of 1.8 m, and a planting density of 4200 plants per 667 m^2^. The test materials of *P. palustre* were obtained locally from Lingshan county and complied with relevant institutional, national, and international guidelines and legislation.

### Determination of plant fresh weight

The yield measurement was conducted in the field on July 22, 2022. A specific number of plants were selected for each treatment to measure their fresh weight. The weighed plants were dried in the field for 1 day, stacked for 1–2 days, and spread out until the moisture content reached approximately 10–15%. Subsequently, a specific number of dried *P. palustre* samples were selected, cut, and ground into powder. The powder samples were then screened using a 200 mesh sieve and utilized for metabolome analysis.

### Determination of soil temperature

The soil temperature in various treatments was measured on May 18th, May 25th, and June 1st, respectively. Measurements were taken at 10:00 am, approximately 20 cm below the soil tillage layer.

### Metabolite extraction

50 mg samples were accurately weighed, and the metabolites were extracted using a 400 µL methanol: water (4:1, v/v) solution with 0.02 mg/mL L-2-chlorophenylalanine as internal standard. The mixture was allowed to settle at − 10 °C and treated by High throughput tissue crusher Wonbio-96c (Shanghai wanbo biotechnology co., LTD) at 50 Hz for 6 min, then followed by ultrasound at 40 kHz for 30 min at 5 °C. The samples were placed at − 20 °C for 30 min to precipitate proteins. After centrifugation at 13,000 × *g* at 4 °C for 15 min, the supernatant was carefully transferred to sample vials for LC–MS/MS analysis.

### UHPLC-MS/MS analysis

The UHPLC-Q Exactive system of Thermo Fisher Scientific was used as the instrument platform for LC–MS analysis. The mass spectrometric data were acquired using a Thermo UHPLC-Q Exactive Mass Spectrometer with an electrospray ionization (ESI) source, which could operate in both positive and negative ion modes. The chromatographic conditions, MS conditions, and data preprocessing and annotation referred to the literature^24^.

### Differential metabolites analysis

The analyses of metabolomics data was performed using the Majorbio Cloud platform (https://cloud.majorbio.com). Principal component analysis (PCA) and orthogonal partial least squares discrimination analysis (OPLS-DA) were conducted using the R package ropls (Version 1.6.2). The significantly differential metabolites (DMs) were selected based on the Variable importance in projection (VIP) obtained from the OPLS-DA model and the *p*-value of student’s t test. Metabolites with VIP > 1, *p* < 0.05 were considered significantly differential metabolites^25^. Venn plots were drawn by VennDiagram (R packages) (Version1.6.20), while KEGG pathway enrichment, heatmap, cluster analysis, VIP analysis, and correlation analysis were carried out using scipy(Python) (Version1.0.0).

### Data analysis

SPSS v.17 software was employed for one-way variance analysis (ANOVA), correlation analysis, and membership function analysis. The data means were analyzed using the Duncan test for statistical significance (*p* ≤ 0.05).

## Results

### Effect of different colors of plastic-film mulching on the fresh weight of *P. palustre*

**Figure 1:**
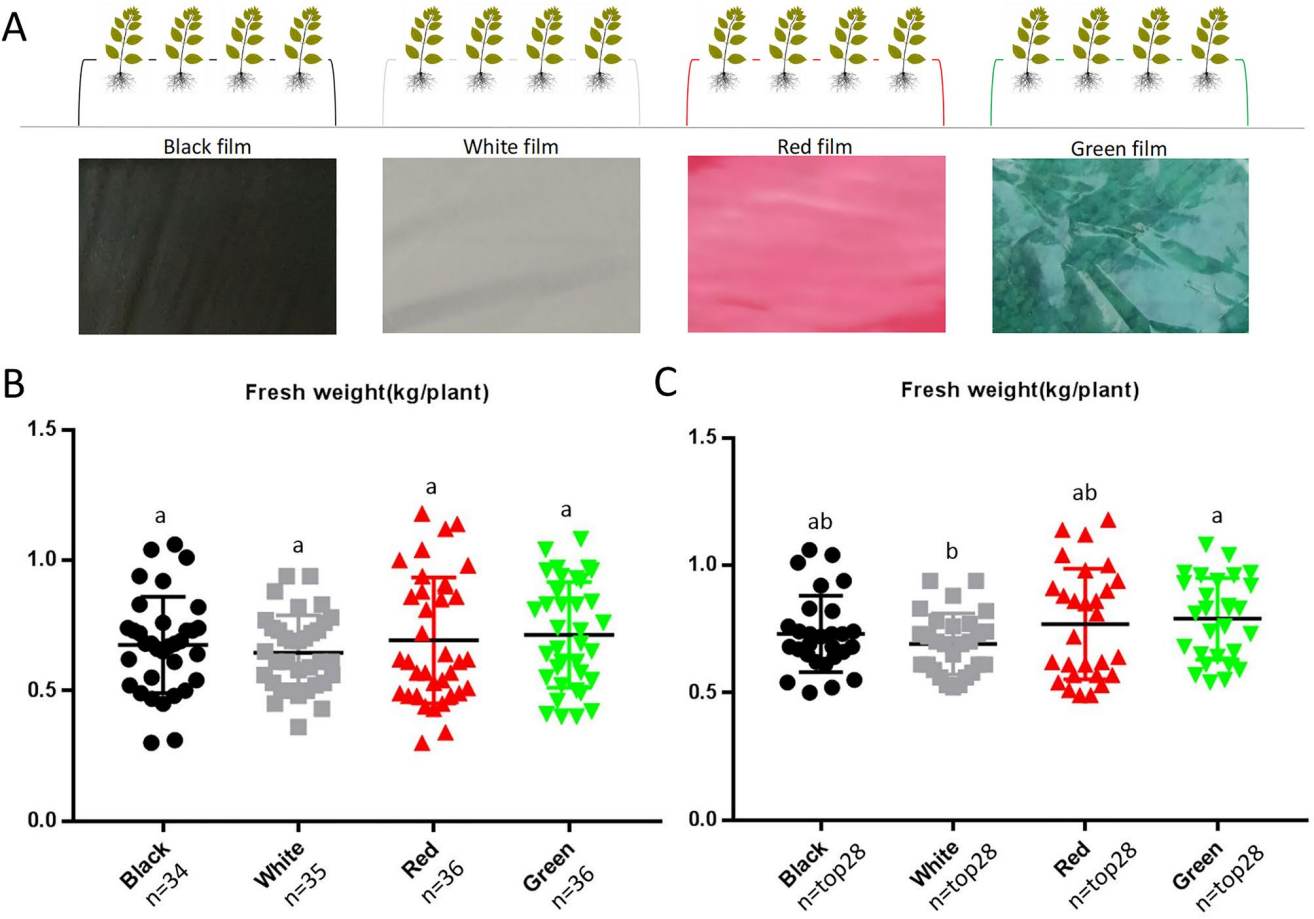
The comparison of fresh weight of individual plant under different treatments. (**A**) The experimental treatment design; (**B**) The fresh weight of individual plant when considering the total number of the samples; (**C**) The fresh weight of individual plant when calculating the data for the top 28 fresh weight per plant. Different letters indicated the significant difference (*p* < 0.05).

In this study, four colors (black, white, red, and green) of plastic-film mulching treatments were adopted (Fig. 1A), and we randomly selected 34, 35, 36, and 36 individual plants from the black film, white film, red film, and green film treatments, respectively. Then the fresh weight per plant was weighed. The results showed that there were no significant differences in the fresh weight per plant between four treatments (n ≥ 34) (Fig. 1B). However, when we calculated the data for the top 28 fresh weight per plant (n = top 28), the fresh weight per plant of the green film treatment was significantly higher than that of the white film treatment (Fig. 1C). Compared with the white film treatment, the fresh weight of the green film treatment increased by 14.15%. In addition, the white film treatment showed the lowest fresh weight per plant, although it was not significantly different from the other two treatments (black and red films).

### Effect of different colors of plastic-film mulching on the soil temperature

**Figure 2:**
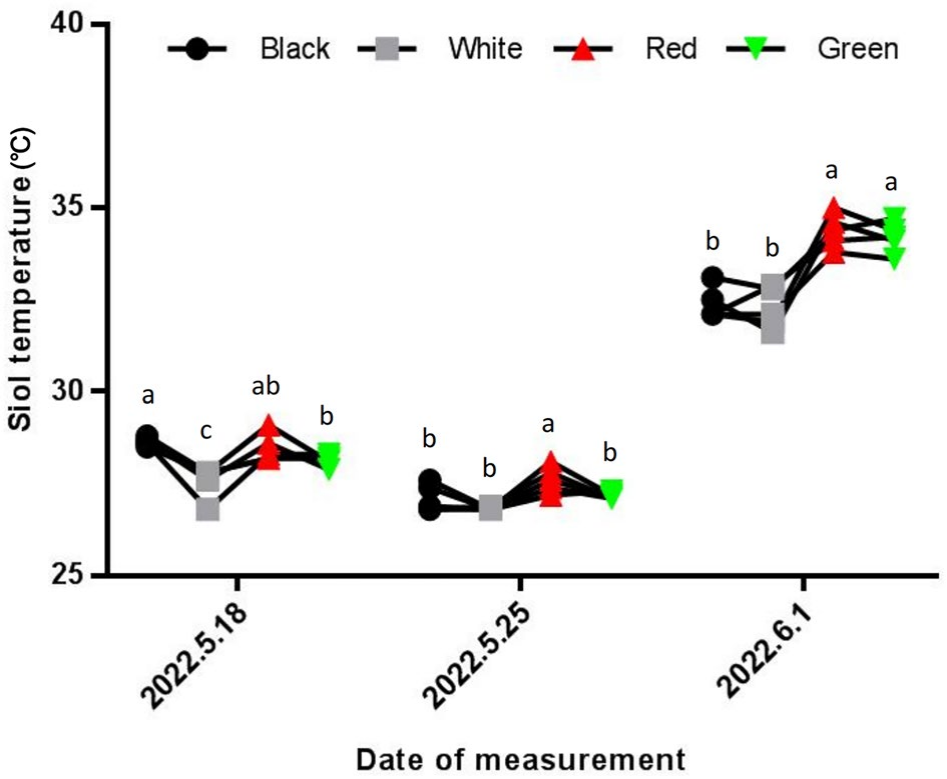
Changes in soil temperature under different treatments at different sampling times.

The soil temperature of different treatments was also determined. As shown in Fig. 2, on May 18, 2022, the soil temperatures in the black and red film treatments were significantly higher than those in the other two treatments. At the second measurement, the highest soil temperature was observed in the red film treatment. The third measurement showed that the highest soil temperatures were found in the red and green film treatments. Based on the results of three temperature measurements, the soil temperature was almost the highest in the red film treatment and lowest in the white film treatment.

### Metabolites of different treatments based on LC–MS

**Figure 3:**
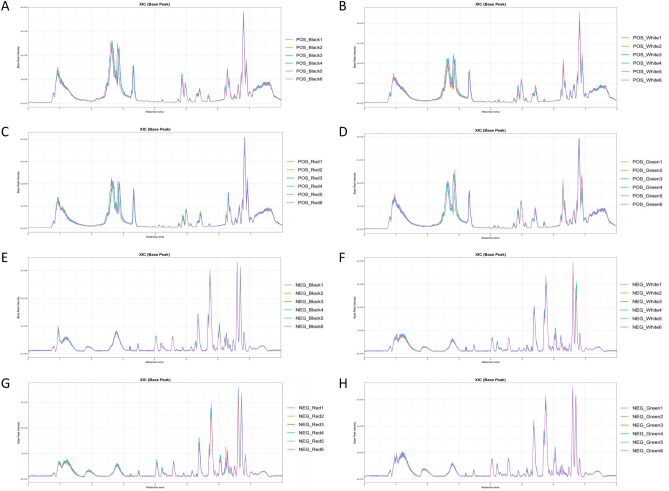
The base peak chromatogram (BPC) of different treatments under positive and negative ion modes. (**A–D**) The base peak chromatogram (BPC) of Black, White, Red, and Green film treatments, respectively under positive ion mode. (**E–H**) The base peak chromatogram (BPC) of Black, White, Red, and Green film treatments, respectively under negative ion mode.

In this study, we extracted and analyzed the metabolites from the whole plant of *P. palustre* with six replicates of each treatment. The base peak chromatogram (BPC) indicted that the obtained data could be used for subsequent analysis (Fig. 3). In total, 1588 and 1604 metabolites were identified under the positive and negative ion scanning modes, respectively (Supplementary Fig. 1). Further analysis of correlation, PCA, Venn, and PLS-DA showed that the data were reliable (Supplementary Fig. 2). KEGG compound classification revealed that these metabolites included several categories: antibiotics, carbohydrates, hormones and transmitters, lipids, nucleic acids, organic acids, peptides, vitamins and cofactors, and so on containing 177 metabolites (Supplementary Fig. 3A; Supplementary Table 1). KEGG pathway exhibited that these metabolites were involved in biosynthesis of other secondary metabolites, amino acid metabolism, lipid metabolism, carbohydrate metabolism and so on (Supplementary Fig. 3B).

### Differential metabolites analysis

**Figure 4:**
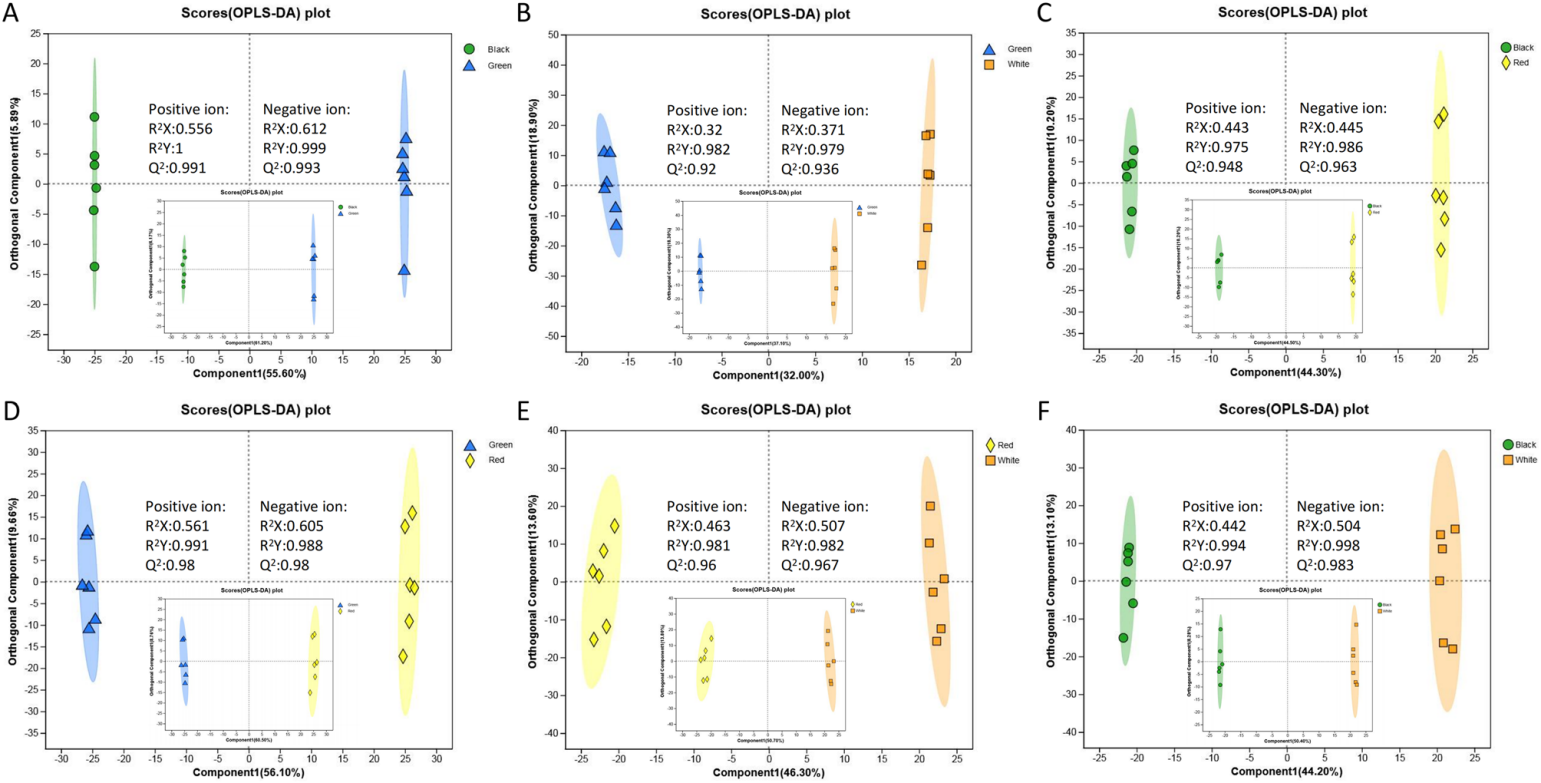
The OPLS-DA plot of different comparison groups. A-F, The OPLS-DA plot of Green_vs_Black, Green_vs_White, Red_vs_Black, Red_vs_Green, Red_vs_White, and White_vs_Black, respectively. The larger and smaller OPLS-DA plots indicated the positive and negative ion modes, respectively.

**Figure 5:**
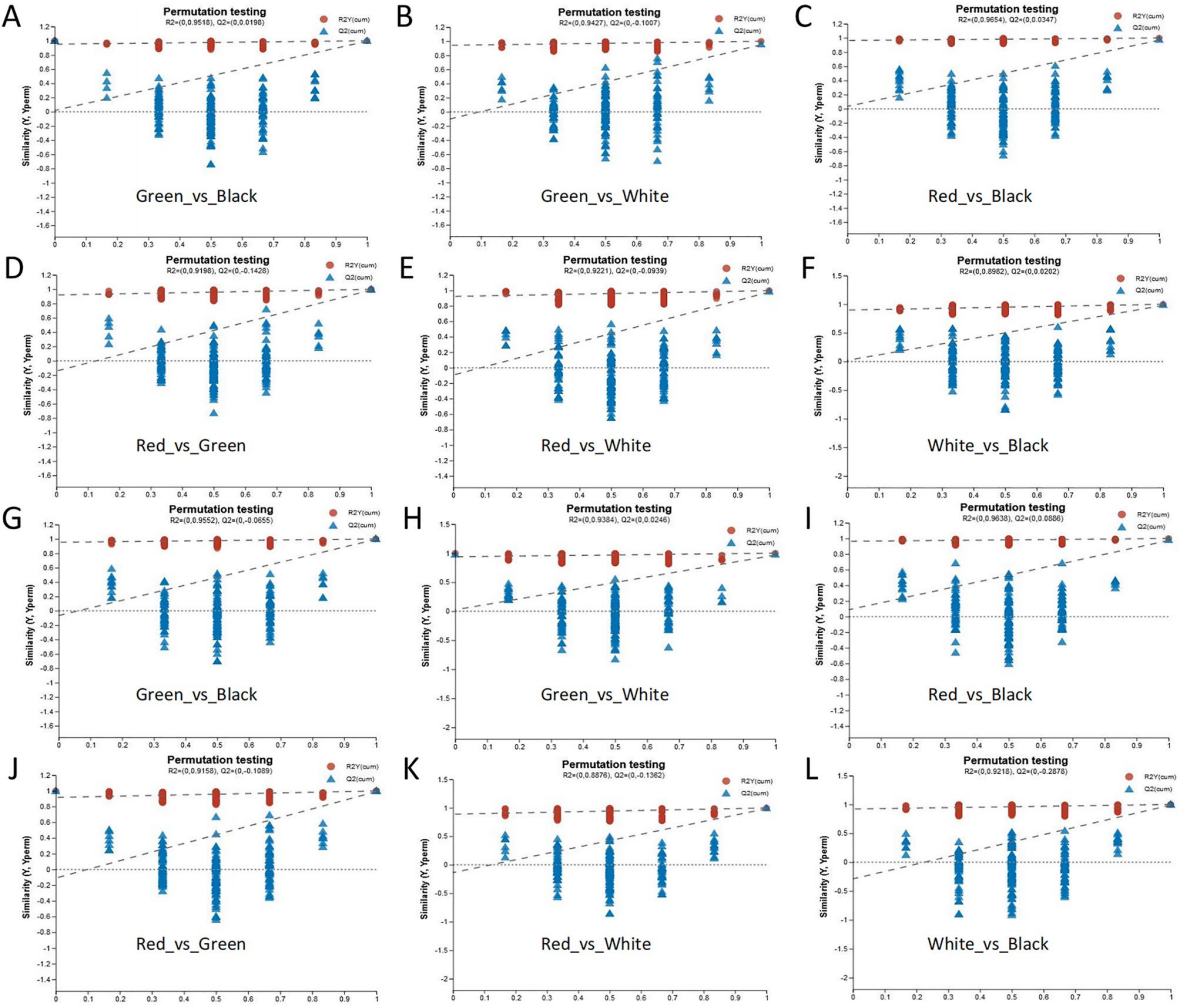
The permutation testing of different comparison groups. (**A–F**) The permutation testing of Green_vs_Black, Green_vs_White, Red_vs_Black, Red_vs_Green, Red_vs_White, and White_vs_Black under positive ion mode, respectively; (**G–L**) The permutation testing of Green_vs_Black, Green_vs_White, Red_vs_Black, Red_vs_Green, Red_vs_White, and White_vs_Black under negative ion mode, respectively.

The different groups could be better distinguished by OPLS-DA analysis (Fig. 4). The model was considered valid when Q^2^ > 50% and R^2^Y-Q^2^ < 0.3^26^, and this criterion was satisfied in both positive and negative ion modes, so the OPLS-DA model fitted well in this study. To ensure the reliability of the results, the OPLS-DA model was also analyzed by permutation testing. As shown in Fig. 5, the Q^2^ intercept of all comparison groups was < 0.05 in both positive and negative ion modes. This indicated that the OPLS-DA model was feasible and the metabolites differed significantly between treatments.

**Figure 6:**
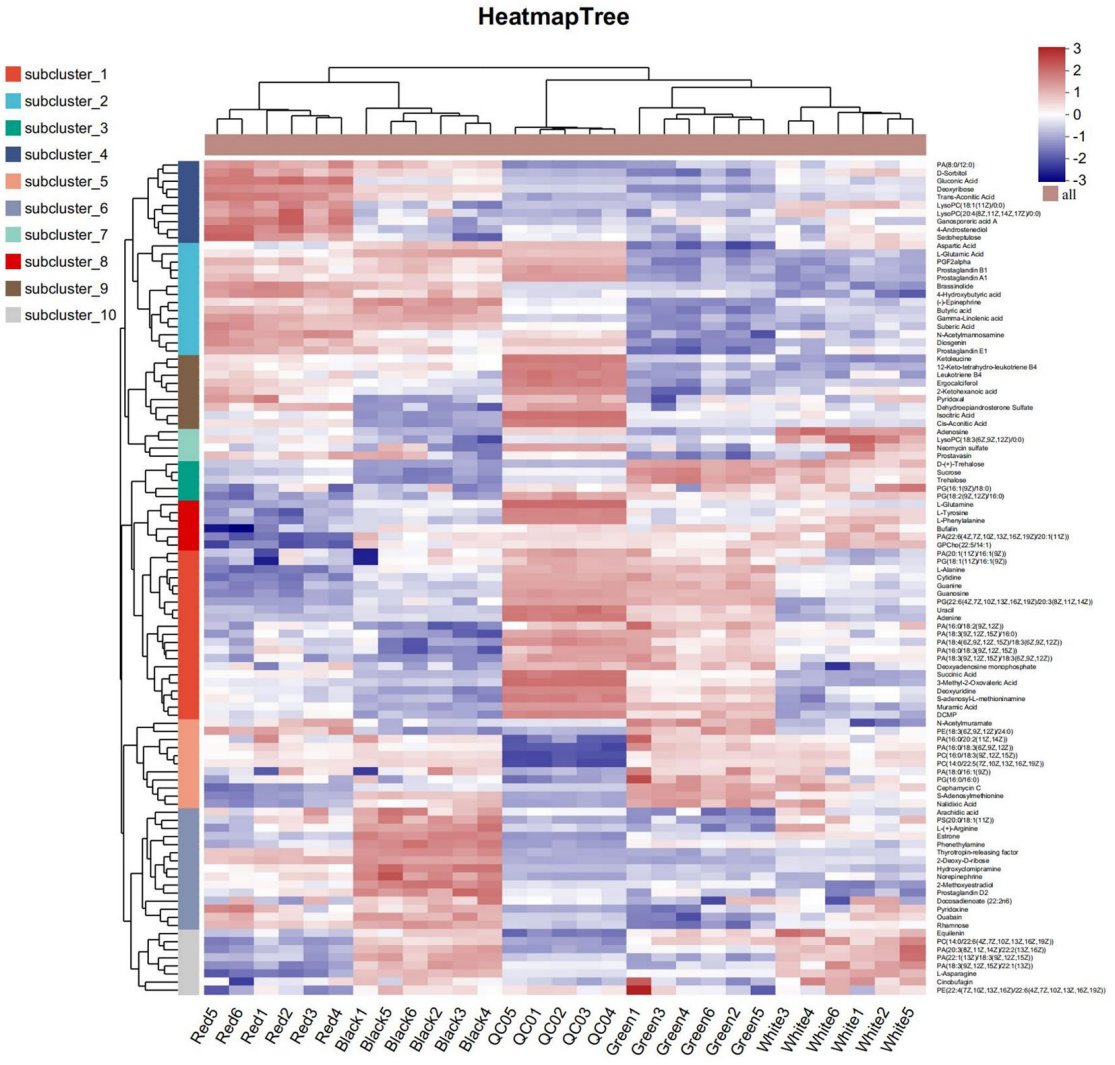
The 103 differential metabolites identified in this study.

Further, according to the VIP > 1 and *p* < 0.05, all the identified metabolites were used for screening the differential metabolites, and the results of the different comparison groups were analyzed. The results showed that a total of 1775 differential metabolites were identified in this study. Of these, there were 867, 821, 775, 743, 810, and 872 differential metabolites were identified in Green_vs_Black, Red_vs_Black, Red_vs_White, Red_vs_Green, Green_vs_White, and White_vs_Black, respectively (Supplementary Fig. 4). Based on these differential metabolites, KEGG compounds classification showed that a total of 103 differential metabolites were identified in this study (Fig. 6). These differential metabolites were classified into different categories, including phospholipids, monosaccharides, oligosaccharides, carboxylic acids, amino acids and so on (Supplementary Table S2). Among these, there were 42, 47, 42, 46, 57, and 39 differential metabolites in Green_vs_Black, Red_vs_Black, Red_vs_White, Red_vs_Green, Green_vs_White, and White_vs_Black, respectively (Supplementary Fig. 5). Further KEGG enrichment analysis revealed that the differential metabolites of different comparison groups were significantly enriched in different metabolic pathways (Supplementary Table 3). For example, in Green_vs_White, a total of 61 differential metabolites were involved in 7 metabolic pathways, which were the highest number of differential metabolites and metabolic pathways significantly enriched. This indicated the greatest difference between the two treatments. Conversely, the differential metabolites in Red_vs_Green were not significantly enriched in any metabolic pathway. Next, we focused on analyzing the differences in the sixteen key metabolites between treatments (Table 1).

**Table 1:** The key differential compound information analyzed in this study.

Metabolite	Formula	Retention time	M/Z	RSD	CAS ID
Rhamnose	C_6_H_12_O_5_	1.3781	163.0602812	0.020849051	3615-41-6
Gluconic acid	C_6_H_12_O_7_	1.0349	195.0502964	0.004865047	526-95-4
2-Deoxy-D-ribose	C_5_H_10_O_4_	2.0608	176.0913636	0.005490203	533-67-5
Deoxyribose	C_5_H_10_O_4_	1.7377	133.0495992	0.013284306	533-67-5
*N*-Acetylmannosamine	C_8_H_15_NO_6_	2.8129	204.0862301	0.02155618	7772-94-3
Trehalose	C_12_H_22_O_11_	1.3023	360.1489397	0.007408219	99-20-7
D-(+)-trehalose	C_12_H_22_O_11_	1.0192	377.0850582	0.004476222	99-20-7
Sucrose	C_12_H_22_O_11_	1.0192	341.1083495	0.003663825	57-50-1
L-alanine	C_3_H_7_NO_2_	1.0558	90.05528102	0.01054852	302-72-7;56-41-7
L-glutamine	C_5_H_10_N_2_O_3_	1.0088	147.0761029	0.004061731	5959-95-5;56-85-9
L-phenylalanine	C_9_H_11_NO_2_	2.8448	166.0859065	0.001548424	63-91-2
L-asparagine	C_4_H_8_N_2_O_3_	1.032	133.0605647	0.012903521	70-47-3
L-(+)-arginine	C_6_H_14_N_4_O_2_	0.9928	175.1185585	0.028341783	74-79-3
L-tyrosine	C_9_H_11_NO_3_	2.1131	180.0657164	0.015312761	60-18-4
Aspartic acid	C_4_H_7_NO_4_	0.9645	132.0292579	0.003841493	1783-96-6;617-45-8;56-84-8
L-Glutamic acid	C_5_H_9_NO_4_	1.0895	146.0449125	0.003989579	6893-26-1;56-86-0

### Monosaccharides

**Figure 7:**
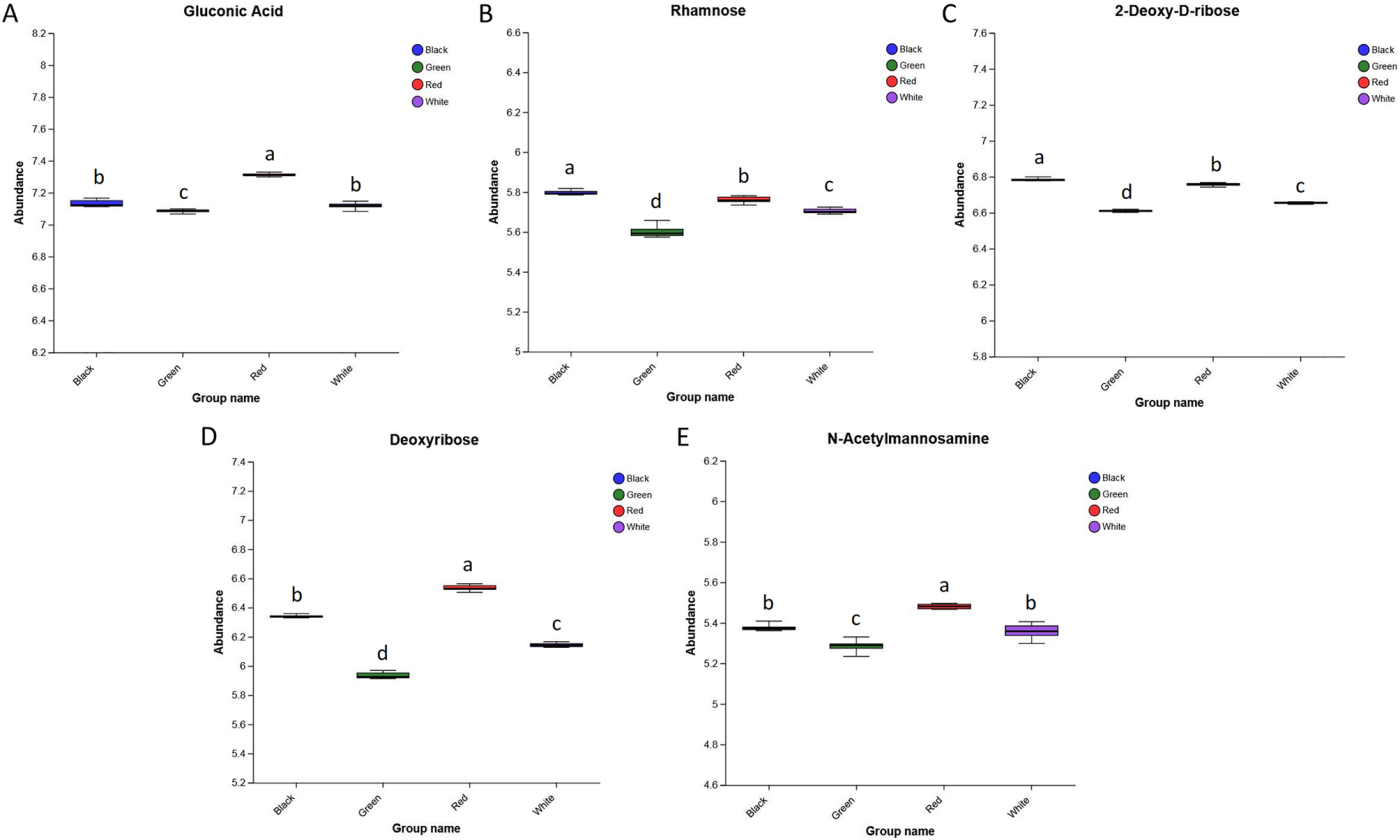
The abundance of differential monosaccharides. (**A**) The expression abundances of Gluconic Acid in four treatments. (**B**) The expression abundances of Rhamnose in four treatments. (**C**) The expression abundances of 2-Deoxy-D-ribose in four treatments. (**D**) The expression abundances of Deoxyribose in four treatments. (**E**) The expression abundances of *N*-Acetylmannosamine in four treatments.

Polysaccharides are one of the most important quality indicators of *P. palustre.* Zhang et al.^27^ found that *P. palustre* polysaccharides consisted of eight monosaccharides including galacturonic acid, glucose, galactose, xylose, mannose, rhamnose, ribose, and glucuronic acid. In this study, five monosaccharides (gluconic acid, rhamnose, 2-deoxy-D-ribose, deoxyribose, and *N*-Acetylmannosamine) related to the quality of *P. palustre* were detected. As shown in Fig. 7, the gluconic acid, deoxyribose, and *N*-Acetylmannosamine in the red film treatment presented the highest abundance compared with the other treatments, meanwhile, the abundances of the five monosaccharides in the red film treatment were significantly higher than those of the green film treatment. It was inferred that the red film treatment could significantly increase the monosaccharide content and might be beneficial to the improvement of the quality of *P. palustre*.

### Oligosaccharides

**Figure 8:**
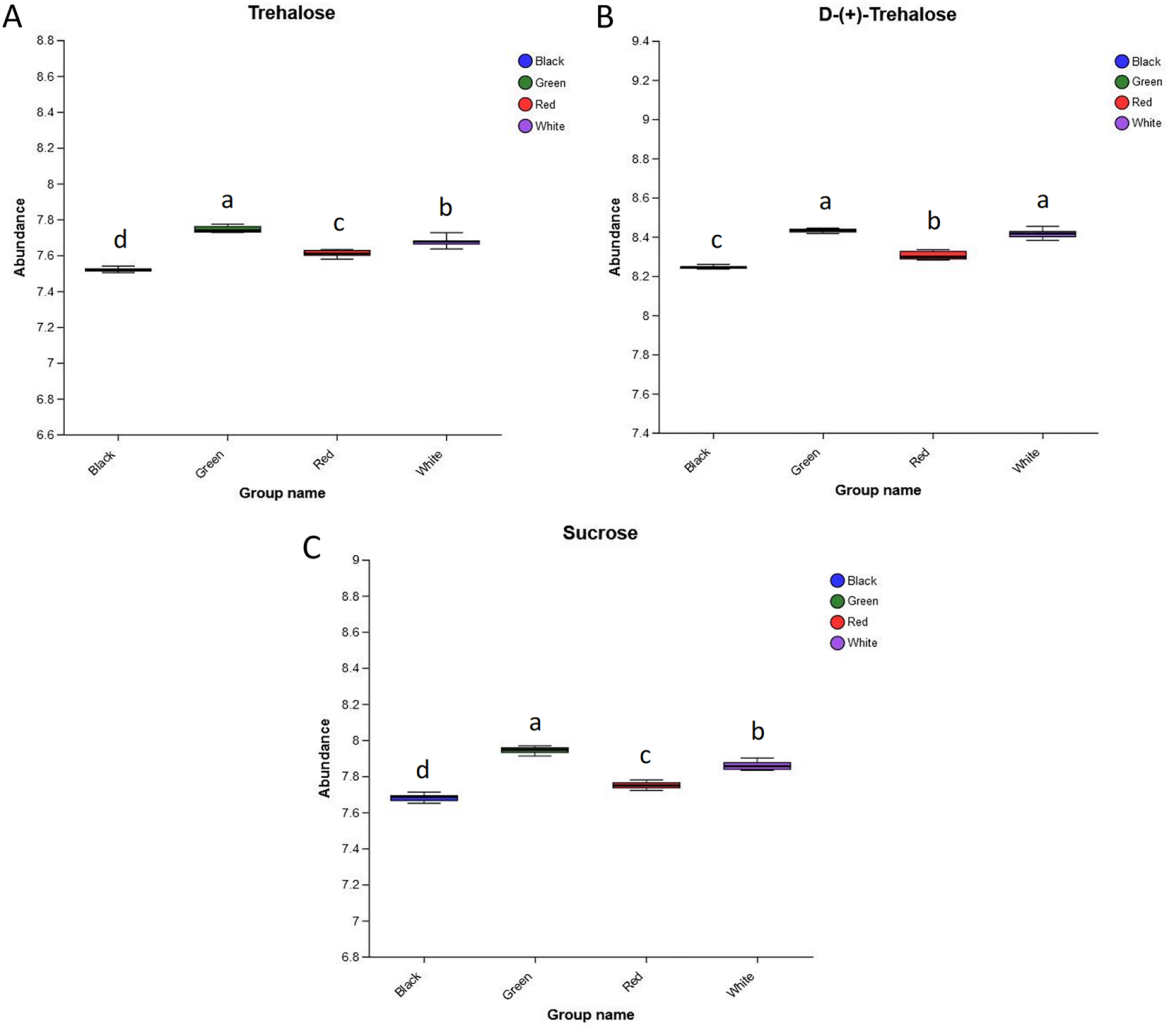
The abundance of differential oligosaccharide. (**A**) The expression abundance of Trehalose in four treatments. (**B**) The expression abundance of D-(+)-trehalose in four treatments. (**C**) The expression abundance of Sucrose in four treatments.

Oligosaccharide refers to any carbohydrate of from 3 to 6 units of simple sugars (monosaccharides). Here, we identified three oligosaccharides including sucrose, trehalose, and D-(+)-trehalose. The abundances of these three oligosaccharides in the black film treatment were significantly lower than those of the other treatments. On the contrary, these three oligosaccharides in the green film treatment exhibited the highest abundance (Fig. 8). Therefore, it was indicated that the green film treatment could promote the content of oligosaccharides of *P. palustre*. Meanwhile, the black film treatment was not conducive to the increase of oligosaccharide content in *P. palustre* in comparison with the other treatments.

### Amino acids

**Figure 9:**
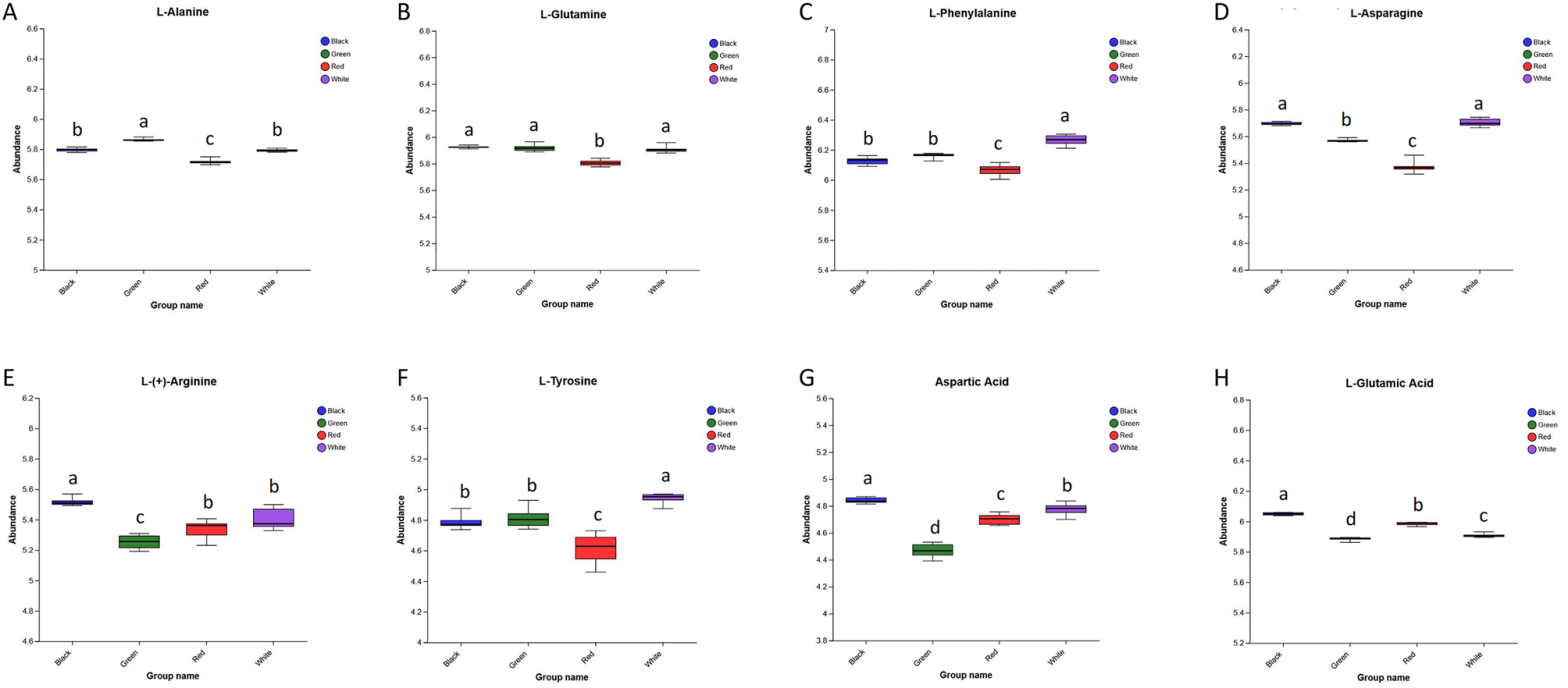
The abundance of differential amino acids. (**A**) The expression abundances of L-alanine in four treatments. (**B**) The expression abundances of L-glutamine in four treatments. (**C**) The expression abundances of L-phenylalanine in four treatments. (**D**) The expression abundances of L-asparagine in four treatments. (**E**) The expression abundances of L-(+)-arginine in four treatments. (**F**) The expression abundances of L-tyrosine in four treatments. (**G**) The expression abundances of Aspartic acid in four treatments. (**H**) The expression abundances of L-glutamic acid in four treatments.

In this study, we detected eight differential amino acids containing L-alanine, L-glutamine, L-phenylalanine, L-asparagine, L-(+)-arginine, L-tyrosine, Aspartic acid, and L-glutamic acid. As could be seen from Fig. 9, the abundances of eight different amino acids in red film treatment were almost the lowest, while those in black film treatment were almost the highest. Alanine, Serine, and Glycine are sweet amino acids in *P. palustre*, and Aspartic acid and Glutamic acid are delicious amino acids^28^. This suggested that the black film treatment was beneficial to the improvement of the nutrition and flavor levels of *P. palustre*.

### Correlation analysis of various indicators

In this study, the correlation analysis on the 18 indicators (except soil temperature) analyzed above were further performed. As shown in Table 2, Rhamnose was positively correlated with 2-Deoxy-D-ribose and negatively correlated with Trehalose and Sucrose (*p* < 0.05). Gluconic Acid was positively correlated with *N*-Acetylmannosamine and Aspartic Acid but negatively correlated with L-Glutamine (*p* < 0.05). 2-Deoxy-D-ribose was negatively correlated with Trehalose, D-(+)-Trehalose, and Sucrose while positively correlated with L-Glutamic Acid (*p* < 0.05). Deoxyribose and L-Phenylalanine were positively correlated with *N*-Acetylmannosamine and Aspartic Acid and L-Tyrosine, respectively, while Sucrose and L-Alanine were negatively correlated with L-Glutamic Acid and Aspartic Acid. L-Glutamic Acid and L-Alanine had a significantly negative correlation with Trehalose and D-(+)-Trehalose and *N*-Acetylmannosamine, respectively, while there was a significant positive correlation between Sucrose, Trehalose, and D-(+)-Trehalose. In addition, there was a significantly positive correlation between *N*-Acetylmannosamine and Aspartic Acid, with a correlation coefficient of 100%.

**Table 2:** Correlation analysis of various indicators under different color plastic-film mulching treatments.

Indicators	Correlation coefficient																
	Rhamnose	Gluconic Acid	2-Deoxy-D-ribose	Deoxyribose	*N*-Acetylmannosamine	Trehalose	D-(+)-Trehalose	Sucrose	L-Alanine	L-Glutamine	L-Phenylalanine	L-Asparagine	L-(+)-Arginine	L-Tyrosine	Aspartic Acid	L-Glutamic Acid	FW
Rhamnose	1																
Gluconic Acid	0.529	1															
2-Deoxy-D-ribose	0.957*	0.592	1														
Deoxyribose	0.874	0.874	0.893	1													
*N*-Acetylmannosamine	0.746	0.951*	0.749	0.967*	1												
Trehalose	− 0.957*	− 0.361	− 0.962*	− 0.760	− 0.575	1											
D-(+)-Trehalose	− 0.906	− 0.467	− 0.980*	− 0.797	− 0.618	0.969*	1										
Sucrose	− 0.974*	− 0.500	− 0.992**	− 0.849	− 0.689	0.988*	0.978*	1									
L-Alanine	− 0.735	− 0.921	− 0.697	− 0.940	− 0.990*	0.536	0.548	0.647	1								
L-Glutamine	− 0.319	− 0.971*	− 0.383	− 0.736	− 0.866	0.128	0.247	0.278	0.846	1							
L-Phenylalanine	− 0.393	− 0.703	− 0.628	− 0.647	− 0.594	0.444	0.649	0.533	.0473	0.628	1						
L-Asparagine	− 0.004	− 0.825	− 0.170	− 0.482	− 0.610	− 0.102	0.096	0.043	0.548	0.900	0.743	1					
L-( +)-Arginine	0.814	− 0.058	0.700	0.430	0.240	− 0.859	− 0.713	− 0.786	− 0.257	0.278	0.070	0.573	1				
L-Tyrosine	− 0.317	− 0.847	− 0.574	− 0.712	− 0.719	0.347	0.547	0.465	0.620	0.808	0.965*	0.875	0.180	1			
Aspartic Acid	0.746	0.951*	0.749	0.967*	1.000*	− 0.575	− 0.618	− 0.689	− 0.990*	− 0.866	− 0.594	− 0.610	0.240	− 0.719	1		
L-Glutamic Acid	0.912	0.394	0.970*	0.757	0.566	− 0.984*	− 0.996*	− 0.980*	− 0.503	− 0.165	− 0.580	− 0.001	0.772	− 0.465	0.566	1	
FW	− 0.409	0.228	− 0.136	− 0.081	− 0.048	0.305	0.062	0.240	0.163	− 0.313	− 0.676	− 0.691	− 0.692	− 0.641	− 0.048	-0.132	1

*Indicated the significant correlation.

Significant values are in [bold].

### Analysis of the membership function values of various indicators

We calculated the D values of the indicators for each treatment using membership functions. The higher the D value, the better the treatment. It could be seen from Table 3, the ranking of different treatments was black > red > white > green, indicating that the black and red film treatments might be more suitable for the cultivation and quality production of *P. palustre* in comparison with the other two treatments.

**Table 3:** Comprehensive evaluation of different treatments.

Treatment	μ(*x*)		D value	Ranking
	μ1	μ2		
Black	0.7580	1.0000	0.8311	1
Green	0.0000	0.1529	0.0462	4
Red	1.0000	0.0000	0.6978	2
White	0.2470	0.7085	0.3864	3

## Discussion

In South China, the temperature in early spring is low and drought in spring often occurs. Plastic-film mulching plays important roles in increasing soil temperature and humidity, reducing weeds and improving crop yield^29^, which has been used in many crops such as corn, peanut, citrus, cucumber, potato, as well as *P. palustre*^1,5–8,30^. Different specifications and colors of mulch have different patterns of projection, absorption and reflection of the spectrum, resulting in different effects on plant absorption of photosensitive pigments, soil environment, light environment and plant growth^31^. For example, compared to the control (no mulch), the yield of fresh ear of corn in transparent mulch, black mulch, and silver mulch increased by 9.25%, 14.47%, and 15.37%, respectively^23^. Similarly, the yield of maize in black mulch and white mulch treatments was significantly higher than that of the control, with a yield increase of 18.77% and 15.28%, respectively, and the yield of black mulch was greater^32^. In this study, the fresh weight of single plant of the green mulch was significantly higher than that of the white mulch and increased by 14.15% in comparison with the white mulch (Fig. 1C). It was indicated that the white film mulch treatment might not be suitable for increasing the yield of *P. palustre* compared with the other treatments. The mulch could change the light environment of the plant by reflecting radiant light^31^. The green mulch reflected green light, which might fulfill the specific light required for *P. palustre* photosynthesis and promote *P. palustre* photosynthesis, resulting in higher yields.

Ngouajio and Ernest^33^ showed that the color of the mulch can determine the amount of light energy and heat obtained, thus directly affecting the soil temperature. Different colors of mulch have different absorption and reflection bands of radiant light, which is reflected in the variability of light transmittance, red film > green film > silver-white film > black film^34^. The greater the light transmittance of the mulch, the more significant the effect of soil warming and the lower the corresponding soil water content^34^. The results of this study were similar to those of Zhong et al.^34^ on colored mulch for strawberry, where the soil temperature of the red film treatment was higher than that of the silver-white film.

Studies have showed that the main components in *P. palustre* mainly includes polysaccharides, flavonoids, phenols, terpenoids, amino acids, etc.^35^. Polysaccharide is one of the important quality indicators of *P. palustre*, and it is also the most abundant (about 20%) and widely used active ingredient in *P. palustre*, with antioxidant, immunomodulatory, antitumor and intestinal flora regulation effects^36–40^. In our study, the abundance of five monosaccharides (gluconic acid, rhamnose, 2-deoxy-D-ribose, deoxyribose, and *N*-Acetylmannosamine) associated with the quality of *P. palustre* was higher under the red film mulch treatment than under the green film mulch treatment, while the abundance of three oligosaccharides (sucrose, trehalose, and D-(+)-trehalose) was highest under the green film mulch treatment (Figs. 7, 8). There were also significant correlations between some monosaccharides and oligosaccharides. For example, rhamnose was negatively correlated with trehalose and sucrose (*p* < 0.05), and 2-deoxy-D-ribose was negatively correlated with sucrose, trehalose, and D-(+)-trehalose (*p* < 0.05) (Table 2). It was indicated that the red film mulch treatment could significantly influence the accumulation of monosaccharides and oligosaccharides and enhance the quality of *P. palustre*. This might be due to the facts that different colors of mulch films affect the light quality, except for black and white-black films, red and green films have higher light transmission at wavelengths of 350–622 nm, and the main light quality of red film treatment is red light, while the main light quality of green film treatment is blue, green, and blue-green light^41^. Similar to our previous study, red light promoted the growth and development of *P. palustre* compared to blue light and increased the content of soluble sugars and pectin in *P. palustre*^13^. This further confirmed that the red film treatment (red light) was beneficial to the quality improvement of *P. palustre*.

Nutritional flavor is also an important quality indicator of *P. palustre*. Amino acids are nutrient substances in *P. palustre*, which have an important influence on the taste of *P. palustre*^42^. Liu and Chen^43^ determined amino acids in artificially cultivated and wild *P. palustre* from Jiangxi, and showed that *P. palustre* contained 18 amino acids, among which glutamic acid and aspartic acid had the highest content. Su et al.^28^ analyzed the differences in amino acid composition and content of *P. palustre* from different sources, and the results showed that all *P. palustre* contained 17 amino acids, with glutamic acid, aspartic acid, and leucine in the top three. In this study, eight differential amino acids were detected, including L-alanine, L-phenylalanine, L-(+)-arginine, L-tyrosine, aspartic acid, L-glutamic acid, L-glutamine and L-asparagine (Fig. 9). Human amino acid taste receptors have a particularly high affinity for glutamate, with other L-amino acids being second to none and D-amino acids being unable to bind^44^. Among these differential amino acids, glutamine is an amino acid generated by amidation of the γ-carboxyl group of glutamate, which is interconvertible with glutamate and asparagine, and the other six amino acids have been detected in previous amino acid determinations of *P. palustre*. Glutamic acid and aspartic acid are the fresh tasting amino acids of *P. palustre* and have important pharmacological effects. Glutamic acid has antioxidant, anti-inflammatory and anti-cancer effects^45–47^, while aspartic acid has anti-tumor and improves diabetic nephropathy^47,48^. Alanine is the sweet amino acid of *P. palustre* that improves diabetic blood glucose levels^49^. Tyrosine has antioxidant and analgesic effects^50,51^. Phenylalanine and arginine, as essential amino acids, are related to the maintenance of nitrogen balance in the body and affect health^52^. Here, eight differential amino acids were almost the highest under the black film mulch treatment, indicating that the black film mulch treatment might be suitable to enhance the nutritional flavor of *P. palustre* compared to other treatments. Differences in soil conditions affect the type and content of amino acids in plants due to the reduced evaporation of soil water under black film mulch, which prevent soil surface caking, maintain soil looseness and increase the oxygen content in the soil, facilitating the development of cactus roots and the activity of soil microorganisms^30,53^. Xiong and Zhu^30^ showed that soil microorganisms were tenfold higher under black film mulch treatment than without film in *P. palustre* cultivation. Therefore, we speculated that the red film treatment might have created a better soil environment, thereby increasing the amino acid content, nutritional flavor level, and potential pharmacological effects of *P. palustre*.

In general, based on the above results, the white film mulch treatment might not be suitable for the cultivation of *P. palustre*. The red film mulch treatment increased the content of monosaccharides, and green film mulch treatment increased the content of oligosaccharides, indicating that red film mulch treatment was beneficial to improve the quality of *P. palustre*. The eight differential amino acids were highest almost when under black film mulch treatment, indicating that the black film mulch treatment was suitable for improving the nutritional flavor of *P. palustre*. Therefore, in the actual cultivation of *P. palustre*, the use of black or red film treatment may be chosen according to the actual needs.

## Conclusions

The white film mulch treatment was not suitable for the cultivation of *P. palustre*. The red film mulch treatment could significantly promote the content of monosaccharides, while the green film mulch treatment could increase the abundance of oligosaccharides. The black film mulch treatment could improve the abundance of amino acids. This study provided a reference for the application of different colors film in the cultivation and production of *P. palustre*, and also provided important data for improving the economic and medicinal value of this species.

### Supplementary Information

Supplementary Figures.

Supplementary Tables.

## Data Availability

The data presented in this study are available on request from the corresponding author.
