# Supplementary material for: Effects of different colors of plastic-film mulching on soil temperature, yield, and metabolites in Platostoma palustre
Source: Sci Rep. 2024 Mar 1;14:5110. doi: 10.1038/s41598-024-55406-w (PMC10907347; doi:10.1038/s41598-024-55406-w)
Supplement: Supplementary file 1 — Supplementary Figures. [file 41598_2024_55406_MOESM1_ESM.docx]

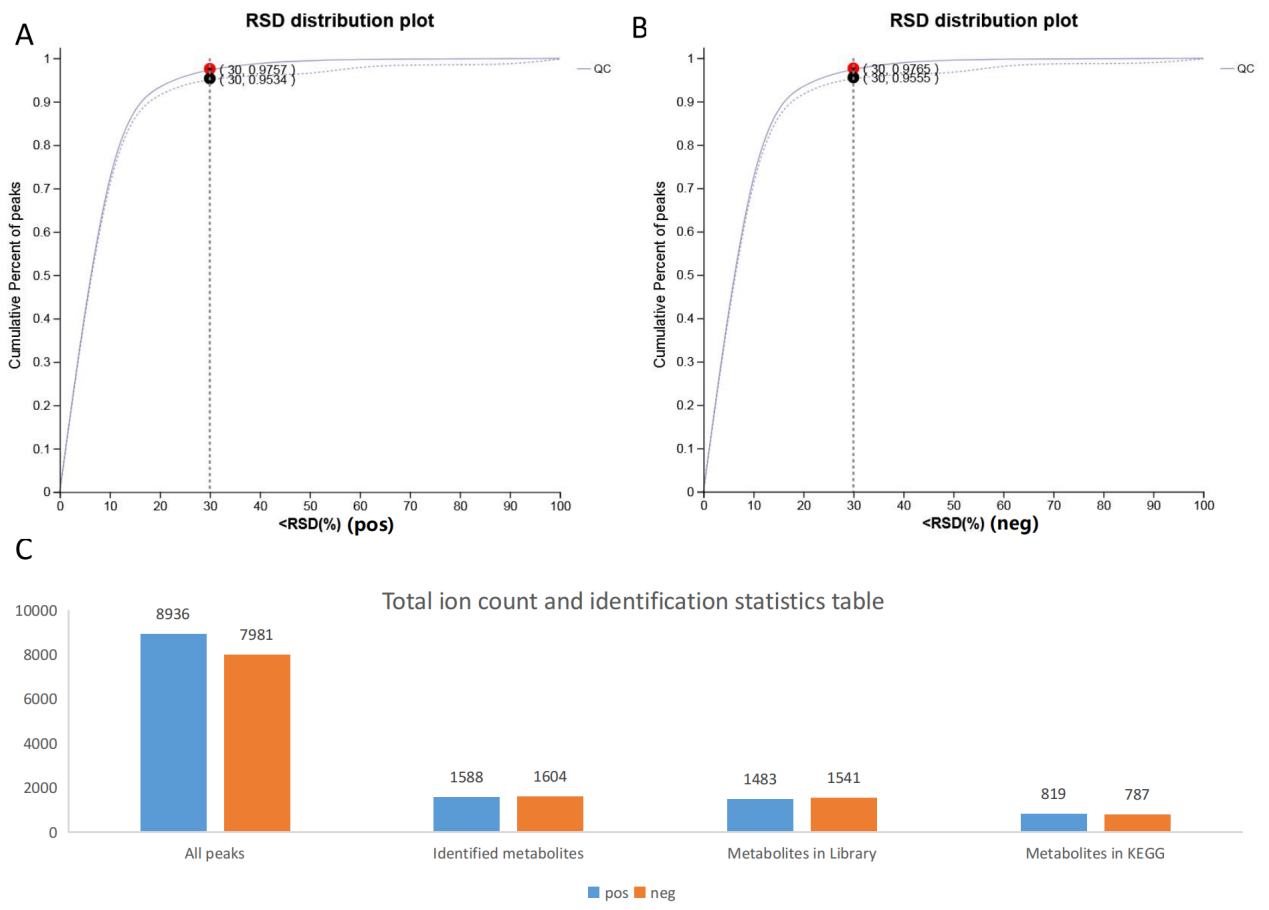


Figure S1 The RSD plot and total ion count and identification statistics. A, The The RSD plot under positive ion mode. B, The The RSD plot under negative ion mode. C, The total ion count and identification statistics.


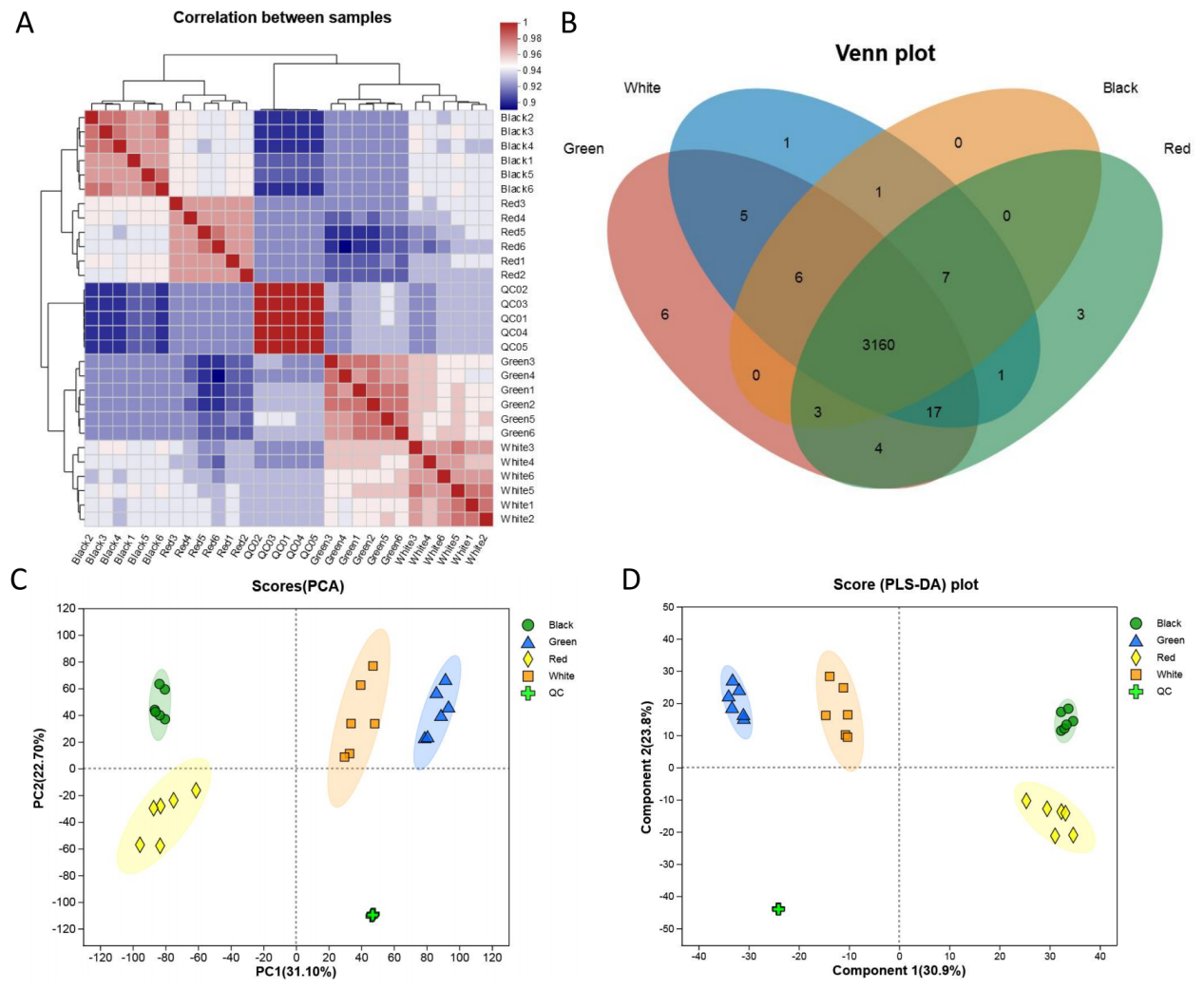


Figure S2 The correlation analysis, Venn plot, PCA, and PLS-DA plot. A-D, The correlation analysis, Venn plot, PCA, and PLS-DA plot, respectively.


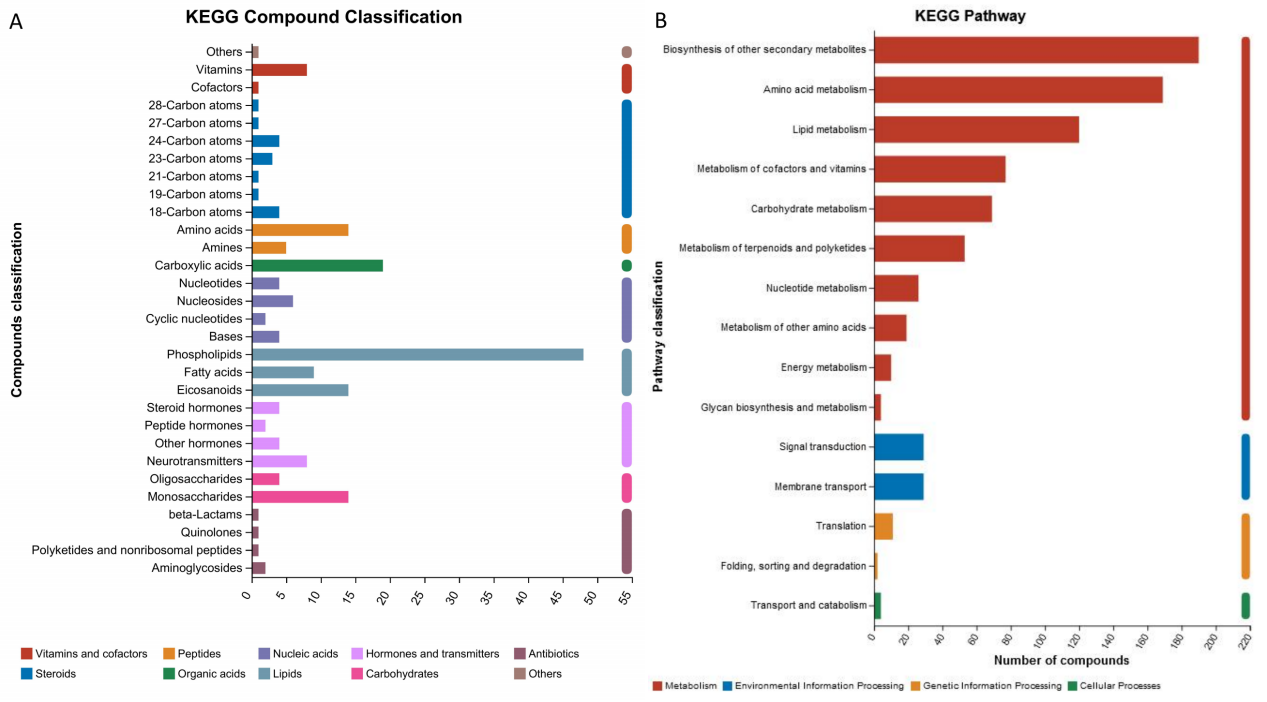
Figure S3 The KEGG compound classification and KEGG pathway.


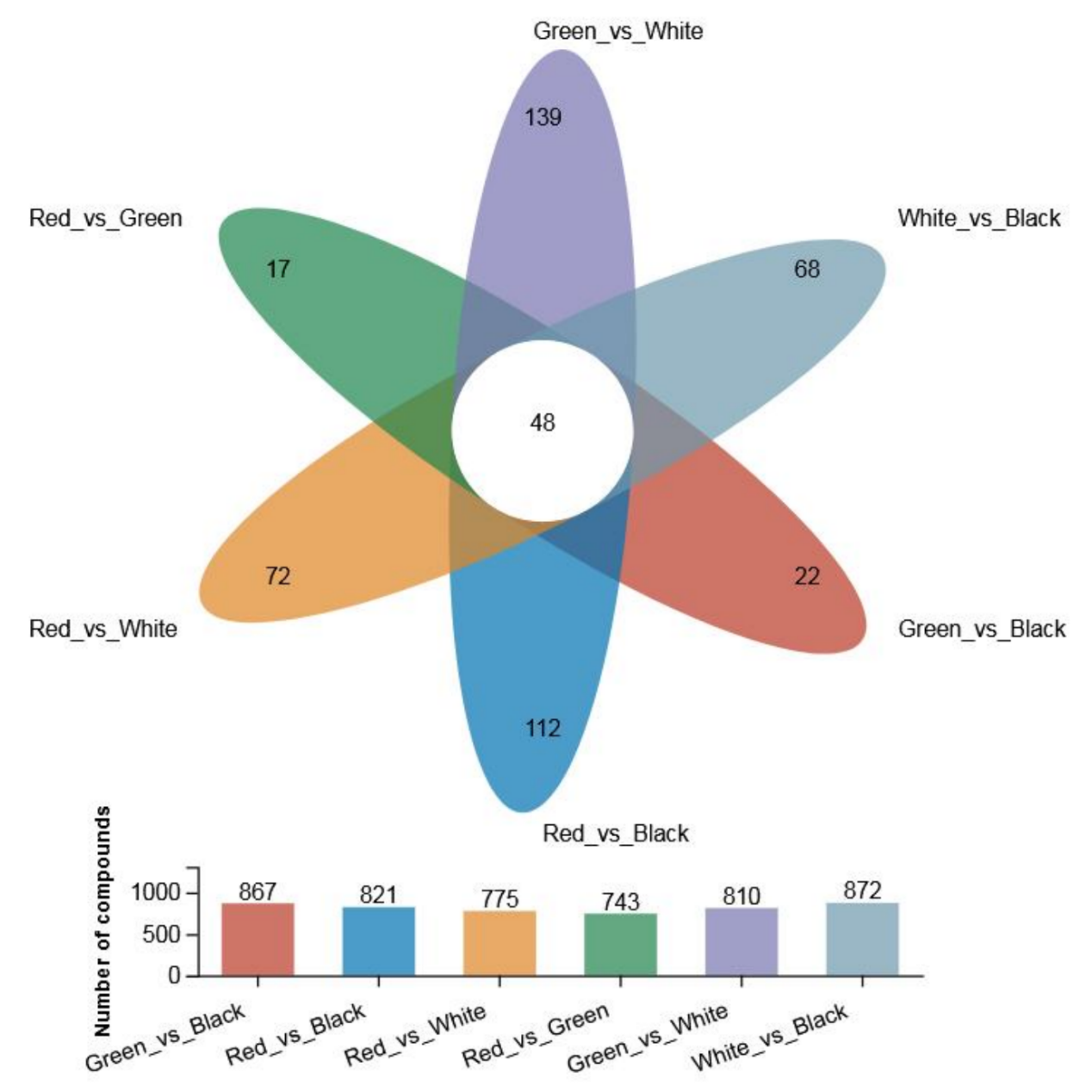


Figure S4 The number of compounds of different comparison groups.


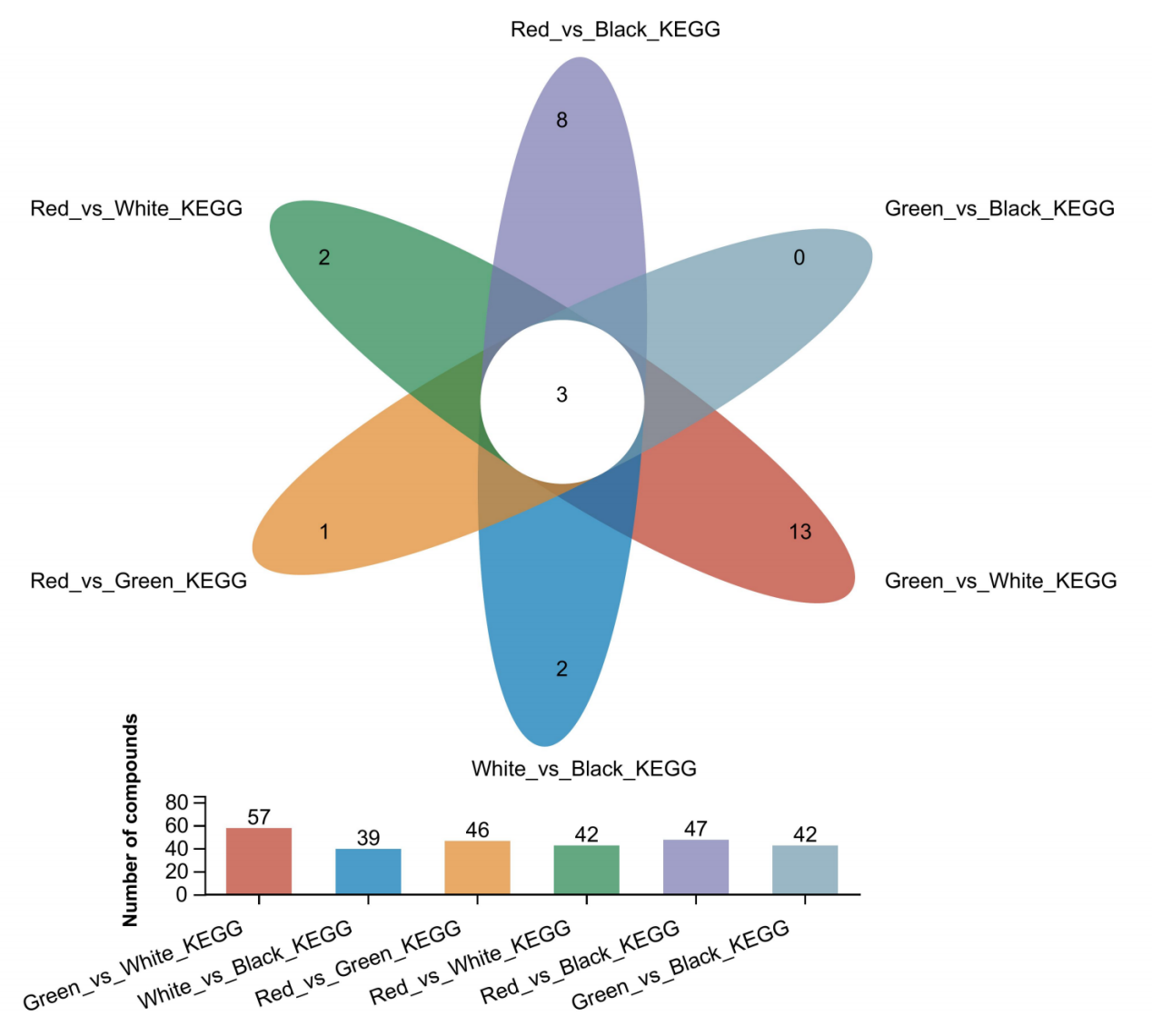


Figure S5 The number of compounds of different comparison groups based on KEGG compound classification.
